# A Microfluidic Deformability Assessment of Pathological Red Blood Cells Flowing in a Hyperbolic Converging Microchannel

**DOI:** 10.3390/mi10100645

**Published:** 2019-09-25

**Authors:** Vera Faustino, Raquel O. Rodrigues, Diana Pinho, Elísio Costa, Alice Santos-Silva, Vasco Miranda, Joana S. Amaral, Rui Lima

**Affiliations:** 1 Center for MicroElectromechanical Systems (CMEMS-UMinho), University of Minho, Campus de Azurém, 4800-058 Guimarães, Portugal; id5778@alunos.uminho.pt (V.F.); d8605@dei.uminho.pt (R.O.R.); 2 MEtRICs, Mechanical Engineering Department, University of Minho, Campus de Azurém, 4800-058 Guimarães, Portugal; rl@dem.uminho.pt; 3 Research Centre in Digitalization and Intelligent Robotics (CeDRI), Instituto Politécnico de Bragança, Campus de Santa Apolónia, 5300-253, Portugal; 4 UCIBIO-REQUINTE, Faculty of Pharmacy of University of Porto, Rua de Jorge Viterbo Ferreira, 4150-755 Porto, Portugal; emcosta@ff.up.pt (E.C.); assilva@ff.up.pt (A.S.-S.); 5 Dialysis Clinic of Gondomar, Rua 5 de Outubro, 4420-086 Gondomar, Portugal; mail@vascomiranda.com; 6 CIMO, Centro de Investigação de Montanha, Instituto Politécnico de Bragança, Campus de Sta. Apolónia, 5300-253 Bragança, Portugal; jamaral@ipb.pt; 7 REQUIMTE-LAQV, Pharmacy Faculty, University of Porto, 4099-002 Porto, Portugal; 8 CEFT, Faculdade de Engenharia da Universidade do Porto (FEUP), R. Dr. Roberto Frias, 4200-465 Porto, Portugal

**Keywords:** microfluidic devices, cell deformability, chronic renal disease, diabetes, red blood cells (RBCs), hyperbolic microchannel, blood on chips

## Abstract

The loss of the red blood cells (RBCs) deformability is related with many human diseases, such as malaria, hereditary spherocytosis, sickle cell disease, or renal diseases. Hence, during the last years, a variety of technologies have been proposed to gain insights into the factors affecting the RBCs deformability and their possible direct association with several blood pathologies. In this work, we present a simple microfluidic tool that provides the assessment of motions and deformations of RBCs of end-stage kidney disease (ESKD) patients, under a well-controlled microenvironment. All of the flow studies were performed within a hyperbolic converging microchannels where single-cell deformability was assessed under a controlled homogeneous extensional flow field. By using a passive microfluidic device, RBCs passing through a hyperbolic-shaped contraction were measured by a high-speed video microscopy system, and the velocities and deformability ratios (DR) calculated. Blood samples from 27 individuals, including seven healthy controls and 20 having ESKD with or without diabetes, were analysed. The obtained data indicates that the proposed device is able to detect changes in DR of the RBCs, allowing for distinguishing the samples from the healthy controls and the patients. Overall, the deformability of ESKD patients with and without diabetes type II is lower in comparison with the RBCs from the healthy controls, with this difference being more evident for the group of ESKD patients with diabetes. RBCs from ESKD patients without diabetes elongate on average 8% less, within the hyperbolic contraction, as compared to healthy controls; whereas, RBCs from ESKD patients with diabetes elongate on average 14% less than the healthy controls. The proposed strategy can be easily transformed into a simple and inexpensive diagnostic microfluidic system to assess blood cells deformability due to the huge progress in image processing and high-speed microvisualization technology.

## 1. Introduction 

Blood is a complex and an extremely information-rich fluid that can be used to diagnose different kinds of blood diseases with multiple biophysical techniques and tools [1,2]. Under normal healthy conditions, the red blood cells (RBCs) comprise about 42% in adult females and 47% in adult males of the total blood volume [3]. As RBCs are the most abundant cells in blood, their deformable properties strongly influence the blood rheological properties, particularly in microvessels with complex geometries and diameters of less than 300 µm [4]. Several research works have found that complex microgeometries, such as contractions [5,6] and bifurcations [2,7,8,9], promote the presence of strong shear and extensional flows that elongate the RBCs without reaching the rupture.

Ever since the RBCs deformability became a potential biomarker for blood diseases, such as malaria [10,11], sickle cell disease [1,12], and diabetes [13,14,15], several techniques have been developed to measure the biomechanical properties of the RBCs. Additionally, there have been several reviews that discuss different kind of experimental methods to measure the RBC deformability [1,2,16,17,18]. The available methods can be divided in two main kinds, i.e., the high-throughput methods that measure high concentrations or diluted suspensions of RBCs, and the single-cell techniques. The most popular high-throughput methods, which include the conventional rotational viscometer [19,20,21], ektacytometer [9,14] and micro-pore filtration assay [9], have been used to measure the blood viscosity and other rheological properties, but they are generally expensive, labor intensive, and do not provide a direct and detailed source of information on the mechanical properties of the RBCs. A recent study that was performed by Sosa et al. [9] has shown that the results from the micro-pore filtration and ektacytometry were often in disagreement, and that neither of them represent the actual blood flow conditions occurring in microvascular networks. Other methods, known as single-cell techniques, which include the micropipette aspiration and optical tweezers, are also extremely popular for measuring the mechanical properties of the RBC membrane [1,13]. However, these techniques also have several drawbacks, such as a low-throughput, labor intensive, and static process. Additionally, it is argued that these methods do not represent the actual RBC deformability that happens during microcirculation [2].

The progress in microfabrication made fabricating microfluidic devices with the ability to directly visualize, measure, and control the motion and deformation of RBCs flowing through constricted [19,22,23,24] and bifurcated microchannels [7,9,24] possible. The distinctive advantage of the microfluidic devices, such as the need of small sample’s volumes and their ability to reproduce more realistic conditions of the microcirculation, have promoted a vast amount of studies on the cell motion and deformability, mainly under the shear flow effect [3,6,16,20,25,26,27]. Some examples are the deformability measurements that were performed under transient high shear stress in sudden constriction channels, [16,28] and in microchannels with dimensions that were comparable to cell size [2,16]. Besides the shear flow effect, the extensional flow and the combination of both can be often found in the microcirculation system, such as in microstenosis and microvascular networks. Hence, during the last decade, several extensional blood flow studies have been performed in cross slot devices [29,30] and in microfluidic devices with hyperbolic channels [31,32,33,34,35,36]. Recent studies that were performed in cross slot devices [37] and sudden constriction channels [26] have shown that cells entrance location and angular orientation strongly affect the cells deformability. On the other hand, extensional flows, where cells are deformed at almost constant strain rates, has been demonstrated to be a microfluidic methodology that is capable of efficiently and accurately probing singe-cell deformability with high throughputs [16,29].

Additionally, the ability of hyperbolic-shaped contraction channels to generate constant strain-rates makes them a promising strategy for measuring RBCs deformability under a well-controlled microenvironment. Taking these advantage into account, the present study investigates the ability of hyperbolic microfluidic channels to measure the deformation and cell motion of RBCs that were obtained from healthy and diseased individuals (having end-stage kidney disease (ESKD), with or without diabetes type II) and exploits the relevance of this flow technique to be used as a viable tool suitable for detecting and diagnosing RBC related diseases.

Chronic kidney disease (CKD) is a pathological condition that results from a gradual, permanent loss of kidney function over time, usually, months to years, which can lead to an end-stage kidney disease (ESKD) [38]. This condition is associated with a decreased quality of life [39], increased hospitalization [39,40], cardiovascular complications, such asangina, left ventricular hypertrophy (LVH), and chronic heart failure, and increased mortality [41,42]. 

The remainder of this paper is organized, as follows: Section 2 comprises several subsections to explain the experimental framework around blood samples, setups used to acquire the data, and methods used to analyze it. Section 3 and Section 4 presents and results and discussion, respectively. 

## 2. Materials and Methods

### 2.1. Patients 

In this study, a total of 20 ESKD patients under online hemodiafiltration (OL-HDF) that voluntary accepted to participate in the study, have been tested. From those, eight additionally showed diabetic nephropathy. Patients were excluded if they: (1) did not accept to participate in the study; (2) were under 18 years old; (3) were cognitively impaired; (4) had a severe speech or hearing impairment; (5) were in the dialysis program for less than three months; and, (6) presented malignancy, autoimmune, inflammatory, or infectious diseases. 

The control group included seven healthy volunteers presenting normal haematological and biochemical values, with no history of renal or inflammatory diseases, and, as far as possible, age- and gender-matched with ESKD patients. The controls did not receive any medication known to interfere with the studied variables. Blood samples (using EDTA as anticoagulant) were drawn from the fasting controls or before the second dialysis session of the week in ESKD patients.

All of the blood samples were obtained from dialysis patients at the hemodialysis clinic of Gondomar, in Porto, Portugal. Informed consent was obtained from all the participants and this study was approved by the clinic’s ethics review board. 

### 2.2. Microfluidic Device, Experimental Setup and Parameters

**Figure 1:**
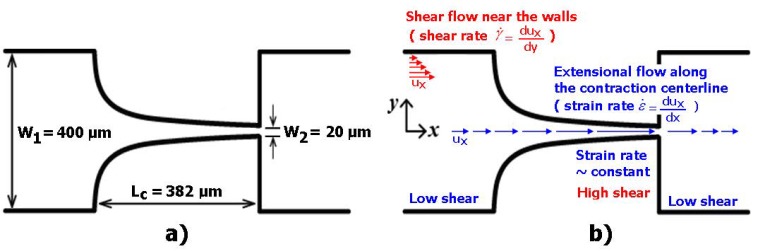
Microfluidic device fabricated in polydimethylsiloxane (PDMS) with a hyperbolic-shaped contraction to assess the of the red blood cells (RBCs) deformability: (**a**) main dimensions; (**b**) flow phenomena happening in this kind of geometry. Adapted with permission from [45].

The polydimethylsiloxane (PDMS) microfluidic devices that were evaluated in this work were fabricated by using a conventional soft-lithographic technique [22]. To perform the deformability assessment, hyperbolic converging microchannels were fabricated with 382 μm of length (L_c_), as well as maximum width of 400 μm (W_1_) and minimum width of 20 μm (W_2_) at the wide and narrow sizes, respectively (cf. Figure 1). This particular geometry corresponds to a hyperbolic contraction with a Hencky strain (*ε_H_*) of ~3. Note that the *ε_H_* can be defined as ln (W_1_/W_2_) [32]. The advantages of the use of this hyperbolic geometry for RBCs screening have already been ascribed in previous studies [43,44]. The hyperbolic contraction geometry was chosen, mainly due to the strong extensional flow that was generated in the middle of the microchannel, which is dominant over the shear flow. The cells by passing through the hyperbolic contraction are submitted to a strong extensional flow, where the velocity almost linearly increases, but the strain rate stays approximately constant. Note that the depth was about 50 μm along the full length of the device. 

Figure 1b also shows the main advantage of using hyperbolic converging microchannels. At the entrance of these kinds of geometries, the RBCs tend to exhibit a linear increase of their velocities and consequently the strain rates within the hyperbolic contractions are close to a constant. This flow phenomenon imposes a homogenous mechanical fluid behaviour to the RBCs and avoids some possible motions (tumbling, twisting, and rolling rotations), often observed in abrupt contractions [26]. Hence, by using hyperbolic converging microchannels, most of the RBCs tend to elongate when they flow through the contraction. It is worth mentioning that RBCs motions, such as tumbling, twisting, and rolling rotations, were never observed during our experiments.

**Figure 2:**
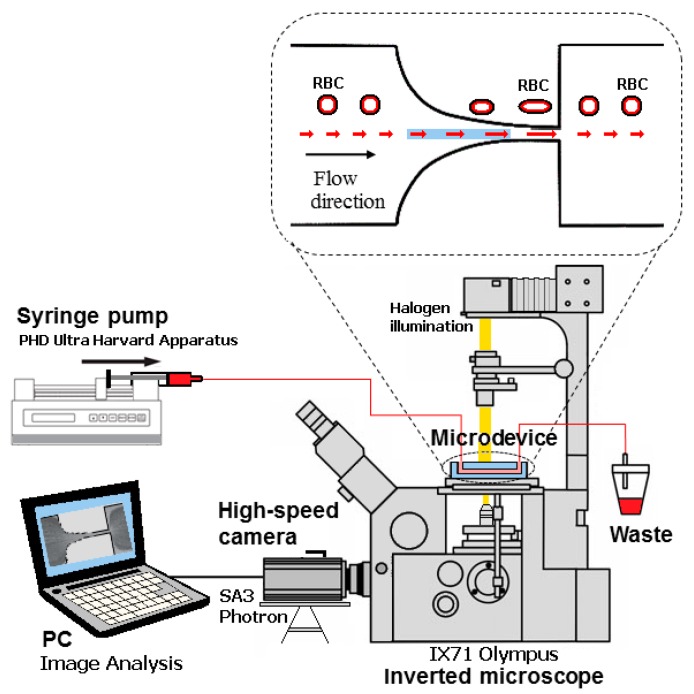
Experimental set-up used to perform the motion and measurements of the RBCs deformability.

The visualization and measurements of the motion of the RBCs were performed by means of a high-speed video microscopy system that includes an inverted microscope (IX71, Olympus, Tokyo, Japan) combined with a high-speed camera (Fastcam SA3, Photron, San Diego, CA, USA). The microfluidic device was placed on the microscope stage and the flow rate of the working fluids was kept constant at 3 μL/min. by using a syringe pump (PHD Ultra, Harvard Apparatus, Holliston, MA, USA) with a 1 mL disposable syringe (Terumo) (Figure 2). For all of the flow measurements, the average shear rate at the hyperbolic contraction region was about 1750 s^−1^. The average or pseudo shear rate was calculated by $\bar{\gamma }=\frac{U}{D_{h}}$, where *U* is the mean velocity of the blood cells that were obtained at the contraction region, and *D_h_* is the hydraulic diameter at the end of the contraction region.

The images of the RBCs flowing through the hyperbolic contraction were captured by the high-speed camera with a frame rate of 3000 frames/s and a shutter speed ratio of 1/75,000 s. These parameters were selected in order to obtain well defined RBCs and avoid possible image distortions that are caused by the high flow velocities at the contraction region. Table 1 shows the most of the relevant experimental parameters that were used to perform the RBCs deformability measurements.

### 2.3. Working Fluids 

**Figure 3:**
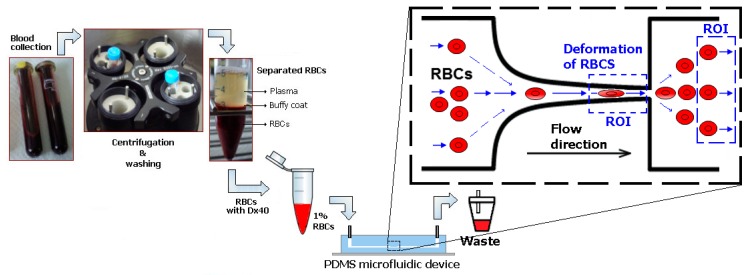
Schematic diagram from blood collection up to the flow microfluidic tests with RBCs. Samples with low hematocrit levels of ~1% were crucial in order to visualize individual RBC flowing through hyperbolic contraction. The ROI regions represent the regions of interest used to analyze the RBCs deformation index.

To perform the RBCs deformability studies, Dextran 40 (Sigma-Aldrich, Saint Louis, MO, USA) at 10% (*w*/*v*) solution containing 1% of haematocrit (Hct 1%, *v*/*v*) of RBCs was used as the working fluid. Briefly, venous blood samples from both patients and healthy donors were collected into 10 mL BD-Vacutainers (BD, Franklin Lakes, NJ, USA) tubes containing ethylenediaminetetraacetic acid (EDTA) to prevent coagulation. The RBCs and buffy coat were separated from the plasma after centrifugation (2500 rpm for 10 min., at 4 °C). The RBCs were then washed with physiological salt solution (PSS) and then centrifuged, with this procedure repeated twice. The RBCs were suspended in Dextran 40 to make several samples with low hematocrit levels of ~1% by volume (cf. Figure 3) to obtain the measurements of individual RBC flowing through hyperbolic contraction. Dextran 40 was used as substitute of the blood plasma, since it prevents not only the sedimentation of the RBCs during the experimental assays, but also the cell clogging phenomenon. All of the analyses were performed within a maximum period of 12 h, with blood samples being hermetically stored at 4 °C until being used in the flow experiments. 

**Figure 4:**
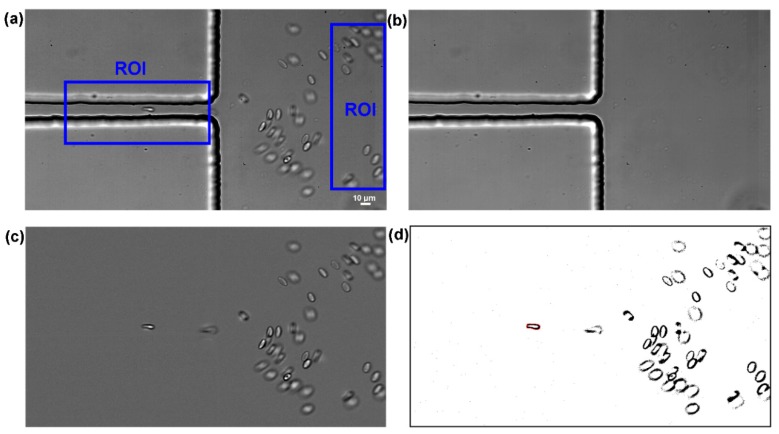
Images analysis sequence: (**a**) original image at the regions of interest (ROI) regions in which moving RBCs as well as microchannel boundaries are visible, (**b**) background image containing only static objects, (**c**) original image after background subtraction showing only moving RBCs, and (**d**) final binary image to perform measurements of the RBCs major and minor axis lengths.

Image analysis was essential to obtain sharper, brighter, and clearer images of the RBCs flowing through the contraction, and to consequently obtain reliable velocity and deformability measurements, at the regions of interest (ROI) in both contraction and expansion regions, where the RBCs deform and recover to their normal circular shape, respectively (see Figure 3a and supplementary video). The first step of this process involves the capture of videos with a resolution of 1024 × 576 pixels at frame intervals of 330 µs at the end of the contraction region. Figure 4a shows a typical obtained image. In order to reduce static artifacts in the images, a background image (Figure 4b) was created from the original stack images, by averaging each pixel over the sequence of static images while using an ImageJ function, called *Z project*, and then subtracted from the stack images. This process eliminates all the static objects including the microchannel walls and some possible attached cells, which resulted in having at the end, only the RBCs of interest (Figure 4c). *Brightness*/*Contrast* adjustment was also applied to enhance the image quality. Finally, the greyscale images were converted to binary images adjusting the threshold level (Figure 4d). At this stage, an *Otsu* threshold method was applied and when required, the level was manually refined. This segmentation process generates objects of interest (RBCs) as black ellipsoidal objects against a white background. At the end, the flowing RBCs in the binary images were measured frame by frame manually, by using *Wand tool* function in ImageJ. The main output results of these measurements were the major and minor axis lengths of the RBCs and the *x-y* coordinates of their centroid.

**Figure 5:**
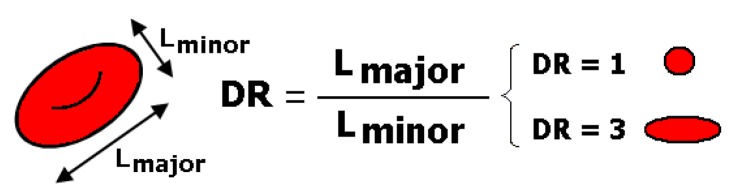
Definition of the deformation ratio, DR = L_major_/L_minor_, where L_major_ and L_minor_ are the major and minor axis lengths of the ellipse best fitted to the cell.

The deformation ratio (DR) of all the measured RBCs was calculated and saved with the cell’s positions, given by their *x-y* coordinates, using the set of data obtained for the cells at the regions of interest (ROI) at both constriction and expansion locations of the microchannel. In this study, DR was defined by the equation that is shown in Figure 5, where L_major_ and L_minor_ refer to the major and minor axis lengths of the RBC, respectively.

Although different automatic methods to track RBCs in microfluidic devices have been reported in the literature [45,46,47,48,49], further improvements still need to be achieved to perform reliable deformability measurements. Hence, in the present study, hundreds of RBCs were manually tracked by using the ImageJ plug-in, MTrackJ. By selecting this method, it is possible to easily track the cells by a centroid based strategy and obtain their centroid position (*x*-*y* coordinates), by carefully tracking individual RBCs and consequently determine their orientations and velocities within the hyperbolic contraction and downstream of the contraction region. In this study, measurements were only performed for the in focus cells flowing from the side, as it is possible to observe in the examples at the supplementary video. In this video, it is also possible to observe a RBC that flows from the top (the biconcave disc shape cell). However, the cells flowing with this orientation were not considered in our deformability measurements.

### 2.4. Statistical Analysis

The statistical analysis was performed by using one-way ANOVA (Microsoft Office Excel, version Office 365 ProPlus). Before performing the ANOVA analysis, the requirements regarding normal distribution were tested by means of the Shapiro–Wilk’s test. In this test, the null hypothesis that the population is normally distributed was accepted since *p* > 0.05. Overall, for the constriction region, we have measured the deformability of 1769 RBCs corresponding to 12 ESKD patients and a total of 736 measured RBCs, eight ESKDD patients and a total of 444 measured RBCs, and seven healthy controls and a total of 589 measured RBCs. All of the statistical tests were performed at a 95% confidence level; differences with *p* < 0.05 were considered to be statistically significant, and were represented as asterisks (*).

## 3. Results and Discussion 

**Figure 6:**
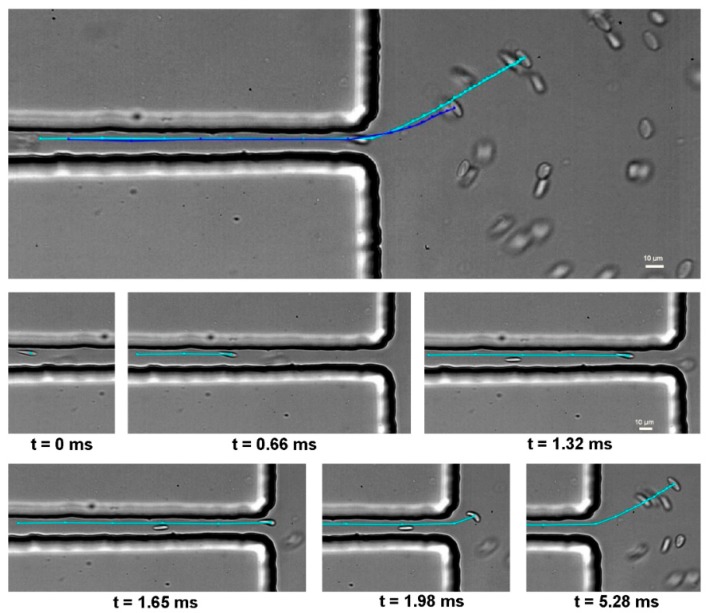
Trajectories of two RBCs flowing within the hyperbolic contraction and downstream of the contraction region (Upper part); detail of a representative trajectory of a RBC flowing near the microchannel wall at different times intervals (Bottom part).

The determination of the RBC velocities plays an essential role in confirming whether the cells are deformed under similar flow conditions. Hence, before the deformability assessment of each sample, velocity measurements were performed and compared. After analyzing the average velocities of each sample at the contraction region, it was decided to compare the RBC deformability for all of the samples having similar flow conditions, i.e., both shear and extensional flows. Figure 6 shows representative RBC trajectories that were manually tracked within the hyperbolic contraction and downstream of the contraction region. 

**Figure 7:**
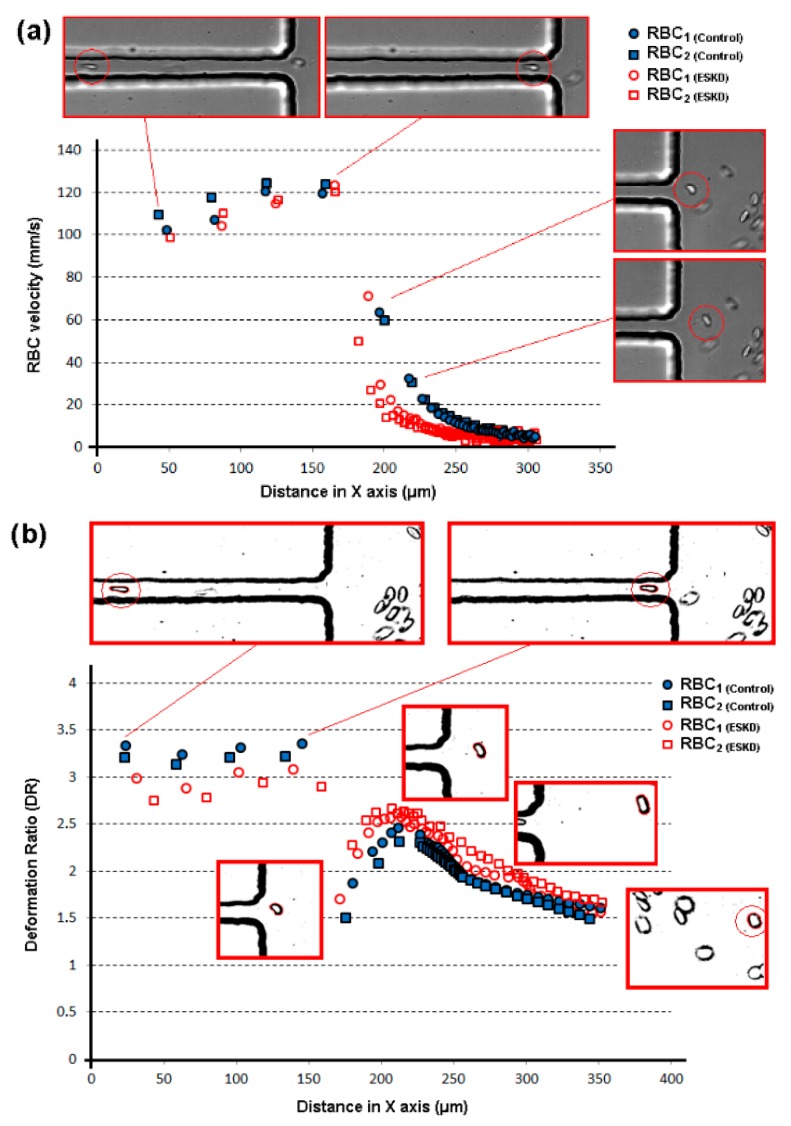
Measurements of RBCs from healthy donors and end-stage kidney disease (ESKD) patients, flowing within the hyperbolic contraction and downstream of the contraction region: (**a**) velocity measurements; (**b**) deformability measurements. The X axis represents the position of the cells centroid flowing through the microchannel.

**Figure 8:**
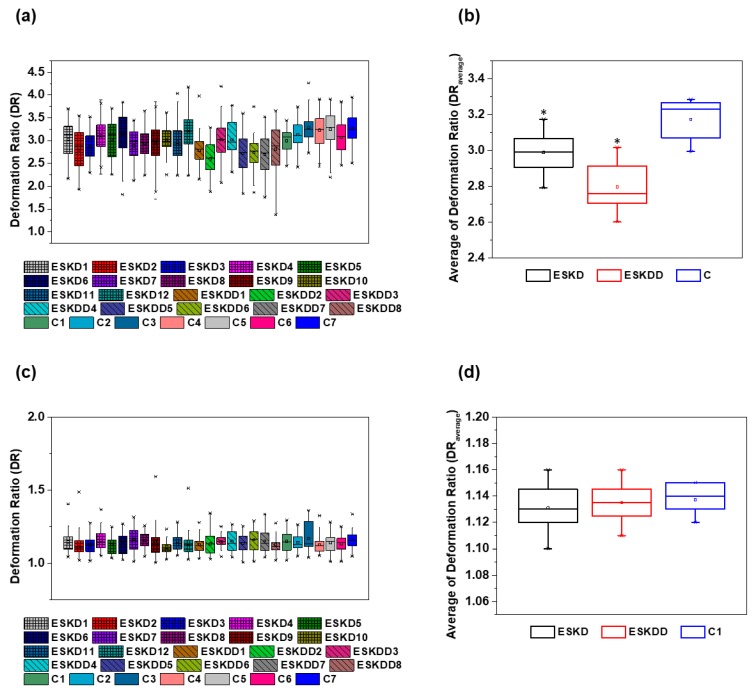
Box plot representation of RBC’s deformation ratio (DR) measured by the proposed microfluidic device: (**a**) DR of individual donors, including ESKD patients, ESKD patients with diabetes and healthy donors (control) in the hyperbolic constriction, (**b**) Average DR of the groups of donors, including ESKD patients, ESKD patients with diabetes and healthy donors (control) in the hyperbolic constriction, (**c**) DR of individual donors, including ESKD patients, ESKD patients with diabetes and healthy donors (control) at the expansion region, (**d**) Average DR of the groups of donors, including ESKD patients, ESKD patients with diabetes and healthy donors (control at the expansion region. The asterisks (*) indicates statistically significant differences (*p* < 0.05) determined by Student’s *t* test.

Figure 7 shows the measurements of the velocity and DR of representative RBCs flowing through the hyperbolic-shaped contraction (ROI region) for both healthy donors and ESKD patients (see also supplementary video). The majority of the RBC velocities tend to slightly increase as they move through the exit of the contraction, and then they suffer a dramatic reduction of their velocities when flowing from the narrow to the wide region of the microchannel (cf. Figure 7a). Overall, the velocities of the RBCs of both control and ESKD patients present a similar qualitative flow behavior at the tested region of the device, which results in a good agreement in the deformability results obtained in all the samples (cf. Figure 7b). However, it should be noted that, quantitatively, the DR results indicate that the deformability of the ESKD RBCs under extensional flow tend to be smaller when compared to the control RBCs (cf. Figure 7b). These latter results are further confirmed with the measurements that were performed with several ESKD patients and healthy individual, as shown in Figure 8. Additionally, during all the flow visualization measurements at constriction region, the RBCs did not show any tumbling and rolling motion, which was mainly due to the uniform and strong extensional flow generated along the hyperbolic-shaped contraction. Note that, under shear flow, it is extremely common to observe RBCs flowing with complex dynamics, such as tumbling and rolling [26,46]. In the present study, the RBCs only exhibited such kind of complex flow motions at the expansion region, due to the dominant shear flow with respect to the extensional flow (cf. supplementary video). Hence, by using the proposed method, when the RBCs enter into the contraction region, they change from a circular to an elliptical shape, with a tendency to become increasingly elongated as they moved through the hyperbolic contraction. This latter flow behavior is possible to observe in Figure 7b. Additionally, in this figure, it can be observed that, at the downstream of the contraction region, the cells start to recover their nearly circular shape, exhibiting a DR that is close to one.

Figure 8 shows the box plot of the deformation ratio (DR) for three different groups, i.e., samples of ESKD patients without diabetes type II (n = 12), samples of ESKD patients with diabetes (n = 8) type II, and samples from healthy donors (n = 7). For each patient sample, more than 60 RBCs with similar flow behavior were individually measured and analyzed at the hyperbolic constriction and recovering channel of the proposed microfluidic device (Figure 8). Additionally, Table 2 shows the data of the average DR and standard deviation (SD) of the RBCs deformation at both the contraction and expansion region for all of the tested samples. Overall, the deformability of ESKD patients (with and without diabetes) measured at the hyperbolic constriction is lower in comparison with the RBCs from the normal healthy controls (*p* < 0.05), as shown in Figure 8b. This difference is more evident when only the group of ESKD patients with diabetes is taken into consideration. For instance, RBCs from ESKD patients without diabetes elongates, on average, 8% less within the hyperbolic contraction when compared to healthy controls, whereas RBCs from ESKD patients with diabetes elongates on average 14% less than the healthy controls (cf. Figure 8b). On the other hand, all of the cells analyzed, both healthy and diseased, have been shown to have a similar DR (nearly to 1, i.e., close to a spherical-shape) at the expansion region of the microchannel (Figure 8c,d), where cells tend to recover to their normal circular shape due to the low shear rate and a negligible strain rate. Therefore, the results from the present study demonstrate that the RBCs DR measured by using the proposed microfluidic device can be considered as a sensitive mechanical biomarker, as it was able to detect changes in DR of the RBCs from patients with different diseases in comparison with healthy ones. Moreover, this study also corroborates other previous research works [13,14], where, by using different deformability measurement techniques, it was shown that elongation of RBCs from patients with diabetes is lower in comparison with the non-diabetic healthy controls.

As previously mentioned, RBCs occupy almost half of the total blood volume and, under healthy conditions, they are highly deformable in order to pass through capillaries with dimensions several times lower than the RBCs size [16]. Hence, it is well known that the RBC deformability plays a crucial role in the rheological properties of blood in microvessels, i.e., the decrease of the RBC deformability might result in an increase of the blood viscosity and, consequently, in an increased tendency for microvascular complications and associated diseases. The results that are presented in this study indicate that the ESKD patients with and without diabetes have a tendency to decrease the RBCs deformability and, as a result, night have a substantial impact in the whole blood viscosity of these patient’s health. This can result in an elicit hemolysis in the capillaries and premature sequestration of RBCs by the reticulo-endothelial system, and altering tissue oxygenation. However, a larger scale study is required to confirm whether the decrease of the RBC deformability contributes to the increase of the blood viscosity, or not.

## 4. Limitations and Future Directions

The primary goal of the present work was to investigate the ability of hyperbolic converging microchannels to be used as an alternative clinical tool that is suitable to detect and diagnose RBC related diseases. To accomplish it, high speed microfluidic studies were performed in a hyperbolic contraction microchannel with a uniform depth of about 50 µm. The selection of this depth was a compromise solution mainly due to the limitation of our high-speed video camera: although, by decreasing the microchannel depth, the orientation of the cells tend to be more stable, the difficulty to measure the RBCs deformability increases due to the extremely high velocities that were generated at this region. The major advantage of this geometrical modification is the ability to use simple automatic methods, which results in a significant increase of the number of cell measurements performed under similar flow conditions. Nevertheless, in this study, we opted for manual measurements to guarantee that only adequate cells were included. 

Additionally, we would like to refer that recently, Schonbrun et al. [50] have shown that by using blue light the hemoglobin absorption makes cells extremely visible and easier to track in microchannels for low shear rates. Although this optical option looks promising, further research needs to be performed regarding the ability to measure blood cells at high shear rates. We consider that a combination of both strategies could result in a promising methodology to preform DR measurement of RBCs with high accuracy. The high-speed camera used due to its cost can be a limitation to consider the technique as a common tool; however, during the last two decades, the cost of this technology has been decreasing in an exponential way, thus we believe that it will be possible to have in a more affordable way a high-speed system in the future. Another way can be the use of compact CCD cameras due its capacity to achieve similar sensitivity and exposure, but in an affordable way. 

## 5. Conclusions

RBCs deformability plays a crucial role in microcirculation and the loss of their deformability can be related to many pathologies. The present study investigated the ability of hyperbolic microfluidic channels to measure the deformation and RBC motion from the blood samples of patients with ESKD (with and without diabetes type II) compared to healthy individuals (used as control) and exploit the relevance of this method to be used as viable clinical tool suitable to detect and diagnose RBC related disease. This study has shown the potential of the proposed device to detect changes in DR of the RBCs from patients with different complications. Overall, the elongation of RBCs from the ESKD patients, with and without diabetes, was lower in comparison with the RBCs from the healthy controls, being the difference more evident for the group of ESKD patients with diabetes. Another important finding was related to the comparison of the cells at the expansion region, where the RBCs have recovered their normal circular shape. At this region, the cells were deformed under low shear rate and negligible strain rate. Under those conditions, we have not found any difference between the ESKD patients and the healthy controls. This latter result indicates that the RBCs need to be submitted to high mechanical stresses to deform the cells and, consequently, to detect different state of blood diseases. 

Additionally, the results that are presented in this study indicate that the ESKD patients with and without diabetes have a tendency to decrease the RBCs deformability, which might have a substantial impact in the whole blood viscosity of these patients. However, a larger scale study would be necessary to confirm whether the decrease of the RBC deformability contributes to the increase of the blood viscosity, or not. Although the proposed microfluidic tool requires further improvements, the results that were obtained from the present study suggest that this technique is able to assure a simple and efficient cell deformability assessment at both physiological and pathological situations. 

## Figures and Tables

**Table 1:** Main experimental parameters used to perform the RBCs deformability measurements.

Main Experimental Parameters	
Maximum width of the microchannel	400 µm
Minimum width of the microchannel	20 µm
Total length of the contraction region	382 µm
Depth of the microchannel	50 µm
Flow rate (syringe of 1 mL)	3 µL/min
Average shear rate	1750 s^−1^
Shear viscosity of the Dextran 40	4.5 × 10^−3^ Pa·s
Density of the Dextran 40	1046 kg/m^3^
Haematocrit of the working fluid	1%
Temperature of the working fluid	22 °C
Magnification (M)	40×
Numerical Aperture (NA)	0.75
Frame rate	3000 frames/s
Exposure time	1/75,000 s

**Table 2:** Average DR and standard deviation (SD) of the flowing RBCs at both contraction and expansion region for each sample.

Blood Samples	Contraction Region DR		Expansion Region DR	
	Average	SD	Average	SD
ESKD1	3.03	0.34	1.12	0.08
ESKD2	2.79	0.36	1.10	0.09
ESKD3	2.86	0.26	1.13	0.06
ESKD4	3.11	0.25	1.14	0.08
ESKD5	3.03	0.39	1.12	0.06
ESKD6	3.15	0.37	1.15	0.07
ESKD7	2.89	0.31	1.15	0.08
ESKD8	2.93	0.26	1.16	0.05
ESKD9	2.94	0.35	1.13	0.10
ESKD10	3.02	0.24	1.10	0.05
ESKD11	2.96	0.32	1.14	0.06
ESKD12	3.17	0.35	1.13	0.08
ESKDD1	2.78	0.25	1.12	0.05
ESKDD2	2.60	0.27	1.13	0.07
ESKDD3	3.01	0.32	1.15	0.05
ESKDD4	3.02	0.32	1.13	0.08
ESKDD5	2.72	0.37	1.14	0.07
ESKDD6	2.74	0.28	1.16	0.08
ESKDD7	2.69	0.34	1.14	0.08
ESKDD8	2.81	0.43	1.11	0.06
C1	2.99	0.21	1.14	0.07
C2	3.12	0.23	1.13	0.06
C3	3.28	0.22	1.13	0.10
C4	3.23	0.27	1.12	0.06
C5	3.24	0.31	1.15	0.07
C6	3.07	0.30	1.14	0.07
C7	3.27	0.27	1.15	0.06

## Supplementary Materials

The following are available online at https://www.mdpi.com/2072-666X/10/10/645/s1, Figure S1: Images from a video where it is possible to observe that the RBCs rotation only happens at the expansion region of the microchannel, and Video S1: Visualization of RBCs flowing within the hyperbolic contraction and downstream of the contraction region.
