# Supplementary material for: A Microfluidic Deformability Assessment of Pathological Red Blood Cells Flowing in a Hyperbolic Converging Microchannel
Source: Micromachines (Basel). 2019 Sep 25;10(10):645. doi: 10.3390/mi10100645 (PMC6843121; doi:10.3390/mi10100645)
Supplement: Supplementary file 1 [file micromachines-10-00645-s001.zip › SupVideo_v2.doc]

##### SUPPLEMENTARY VIDEO ONLINE

**Supplementary Video**

Visualization of red blood cells (RBCs) flowing within the hyperbolic contraction and downstream of the contraction region, i.e., at the expansion region. In this work, measurements were only performed for the in focus cells flowing from the side as it is possible to observe an example of a RBC that is partially tracked. In this video, it is also possible to observe a RBC flowing from the top (the biconcave disc shape cell). However, the cells flowing with this orientation were not considered in our deformability measurements. The original video was recorded by a high speed camera at 3000 frames/s.

**Figure 1**: Images from a video where it is possible to observe that the rotation only happens at the expansion region of the microchannel.
